# Genetic Inactivation of *Notch1* Synergizes with Loss of *Trp53* to Induce Tumor Formation in the Adult Mouse Forebrain

**DOI:** 10.3390/cancers14215409

**Published:** 2022-11-02

**Authors:** Elena Parmigiani, Claudio Giachino

**Affiliations:** Embryology and Stem Cell Biology, Department of Biomedicine, University of Basel, Mattenstrasse 28, 4058 Basel, Switzerland

**Keywords:** Notch signaling, brain tumor, Rbpj, neural progenitor, forebrain, tumor suppressor

## Abstract

**Simple Summary:**

Notch signaling plays a context-dependent role in multiple cancer types by either promoting or suppressing tumor development. The role of the Notch receptors in the formation of brain tumors remains controversial. By exploiting conditional genetics and lineage tracing approaches to study unperturbed solid tumor growth in vivo, we uncover a tumor suppressor function for the Notch1 receptor in the forebrain and show that p53 and Notch1 cooperate to inhibit tumor formation.

**Abstract:**

Simultaneous genetic inactivation of the key Notch signaling mediator RBP-Jk and p53 leads to the formation of forebrain tumors in mice, suggesting a tumor suppressor role of the Notch pathway in this context. However, the contribution of individual Notch receptors to the tumor-suppressive activity of Notch signaling in the brain remains elusive. Here, we show that simultaneous *Notch1* and *Notch2* deletion, similar to complete ablation of canonical Notch signaling by *Rbpj* inactivation, cooperates with *Trp53* deletion to promote tumor growth in the adult forebrain. We also demonstrate that inactivation of *Notch1* and *Trp53* in cells with active Notch signaling is sufficient to induce brain tumor or hyperplasia formation. Analysis of tumor location suggests a multifocal origin and shows that ventral forebrain regions and olfactory bulbs are the most affected sites. Hence, Notch1 cooperates with p53 to repress malignant transformation in the adult mouse forebrain.

## 1. Introduction

The Notch signaling pathway plays pleiotropic roles during tissue development and tumor formation [1]. Depending on the cellular context and tissue type, Notch signaling can function either as an oncogene or a tumor suppressor [1,2]. This dual function of Notch in the regulation of tumorigenesis has, in some instances, been observed even within the same organ, such as in the lung or the hematopoietic system [3,4,5].

Opposing oncogenic and tumor-suppressive roles of Notch have also been described for the central nervous system (CNS) in mammals [4,6]. Notch signaling is fundamental for the maintenance of neural stem cell (NSC) identity and for the regulation of cell fate decisions in the CNS [7,8]. The Notch1 and Notch2 receptors, the indispensable mediator of canonical Notch signaling RBP-Jk, and the Notch downstream targets of the *Hes/Hey* gene family are all essential in NSCs [9,10,11,12,13,14,15,16,17,18,19]. Despite the stem cell-promoting activity of Notch, which certainly plays roles in Notch oncogenic function in various forms of primary CNS cancers, Notch signaling can also have a tumor-suppressive function linked to the regulation of mitotic quiescence and immune evasion in the brain [4,6,20]. This dual role of Notch is likely related to the vast genomic and epigenomic heterogeneity of CNS tumors [21,22,23,24,25]. Decoding the context dependent functions of Notch signaling is fundamental in order to develop more effective strategies to treat primary brain malignancies.

Reduced Notch signaling activity associated with increased aggressiveness and worse prognosis has been observed in subtypes of glioma and of CNS primitive neuroectodermal tumors [21,23,26,27,28,29,30,31]. Inactivating mutations in Notch pathway components, particularly in the *NOTCH1* and *NOTCH2* receptor and *RBPJ* genes, have been detected in isocitrate dehydrogenase (IDH) mutant gliomas [21,23,26,27,29,32]. In support of these observations in human brain tumor subtypes, simultaneous genetic inactivation of *Notch1* and *Notch2* or *Rbpj* accelerates the growth of PDGF-driven gliomas in mice [20,28]. *TP53* mutations and elevated PDGF signaling are common in human proneural glioma, a transcriptional subtype where Notch signaling can act as a tumor suppressor [24,28]. Since deletion of *Trp53* predisposes to hyperplasia formation but does not induce tumor development in the murine brain, it can be exploited in combination with inhibition of Notch signaling components to reveal their tumor suppressive activity [28]. Strikingly, genetic deletion of *Rbpj* together with *Trp53* induces aggressive, de novo forebrain tumors even in the absence of PDGF signal activation [28]. However, it is not known whether inactivation of *Notch1* and *Notch2* with *Trp53* can induce brain tumor formation. Moreover, the relative contribution of Notch1 and Notch2 receptors to the tumor-suppressive activity of Notch signaling in this context remains elusive. Here, by exploiting long-term genetic lineage tracing approaches to study unperturbed solid tumor growth in vivo, we show that the inactivation of *Notch1* and *Trp53* in Notch signaling-active cells is sufficient to induce tumor or hyperplasia formation in the ventral forebrain and olfactory bulbs (OBs).

## 2. Materials and Methods

### 2.1. Animals

*Hes5::CreER^T2^*, floxed *Rbpj,* floxed *Trp53,* floxed *Notch1,* floxed *Notch2,* and *Rosa-CAG::GFP* mice have been described previously [16,33,34,35,36,37]. Young adult mice between 8 and 10 weeks of age were used for the experiments. Mice were maintained on a 12 h day/night cycle with adequate food and water under SPF conditions and according to institutional regulations under license numbers 2689, 2538, and 2537, and all experiments were approved by the ethics commission of the Kantonales Veterinäramt Basel-Stadt, Basel, Switzerland.

### 2.2. Tamoxifen Administration

Stock solutions of Tamoxifen (TAM, Sigma-Aldrich, St Louis, MO, USA ) were prepared at a concentration of 20 mg/mL in corn oil (Sigma-Aldrich). Adult mice (8–10 weeks of age) were injected intraperitoneal with TAM once per day for 5 consecutive days at a dose of 2 mg/day. Animals were killed 10 months after the last injection, or when they developed symptoms of brain tumor formation (lethargy, poor grooming, weight loss, or macrocephaly), and the brains were prepared for immunohistochemistry as described below.

### 2.3. Tissue Preparation and Immunohistochemistry

For histology, mice were deeply anaesthetized by injection of a ketamine/xylazine/acepromazine solution (130 mg, 26 mg and 4 mg/kg body weight, respectively) and perfused with ice-cold 0.9% saline solution followed by ice-cold 4% paraformaldehyde (PFA) solution in 0.1 M phosphate buffer (PB). Brains were post-fixed with 4% PFA overnight, washed in PB, cryoprotected in a 30% sucrose solution in 0.1 M PB for 48 h, embedded and frozen in OCT (TissueTEK). Free floating coronal sections (30 μm) were collected in multi-well dishes (Corning) and stored at −20 °C in anti-freeze solution until use.

For immunostaining, sections were incubated overnight at 4 °C with the primary antibody diluted in blocking solution of 2% normal donkey serum (Jackson ImmunoResearch), 0.5% Triton X-100 in phosphate-buffered saline (PBS). Sections were washed three times in PBS and incubated at room temperature for 1 h with the corresponding secondary antibodies in blocking solution. When necessary, sections were counter-stained with DAPI (1 μg/mL). For PCNA detection, antigen was recovered at 80 °C for 20 min in Sodium Citrate solution (10 mM, pH 6). Stained sections were mounted on Superfrost glass slides (Thermo Scientific, Waltham, MA, USA), embedded in mounting medium containing diazabicyclo-octane (DABCO, Sigma-Aldrich) as an anti-fading agent and visualized using a Zeiss Observer.Z1 equipped with Apotome.

Tumors were defined as discernible masses (increased cellularity based on DAPI staining) of proliferating (PCNA^+^) cells that were spanning at least 2 consecutive sections in a series of sections spaced 360 μm apart. Hyperplasias were defined as areas containing increased numbers of diffuse, highly proliferative PCNA^+^ cells, with only a limited increase in cellularity (DAPI staining).

The primary and secondary antibodies used are listed below: rabbit anti-PCNA (clone D3H8P, Cell Signaling, Cat# 13110); goat anti-SOX2 (R&D, Cat# AF2018); rabbit anti-OLIG2 (Merck Millipore, Cat# AB9610); rabbit anti-GFAP ( Sigma-Aldrich, Cat# G9269); goat anti-DCX (Santa Cruz Biotechnology, Cat# sc-8066); rabbit anti-RBP-Jk (Cell Signaling, Cat# 5313); chicken anti-GFP (Aves labs, Cat# GFP-1020); sheep anti-GFP (AbD Serotec/Biorad, Cat# 4745–1051); rabbit anti-GFP (Invitrogen, Cat# A11122); donkey Alexa488/Cy3/Alexa647 conjugated anti-rabbit, chicken, goat, and sheep secondary antibodies (Jackson ImmunoResearch)**.** Anti-GFP antibodies were detected with appropriate Alexa488-conjugated secondary antibodies. All other primary antibodies were detected with Cy3- or Alexa647-conjugated secondary antibodies and the images were pseudo-colored in green in some of the figures for visualization purpose.

## 3. Results

### 3.1. Combined Deletion of Notch1/Notch2 and Trp53 in Hes5^+^ Cells Leads to the Formation of Forebrain Tumors in Adult Mice

**Figure 1:**
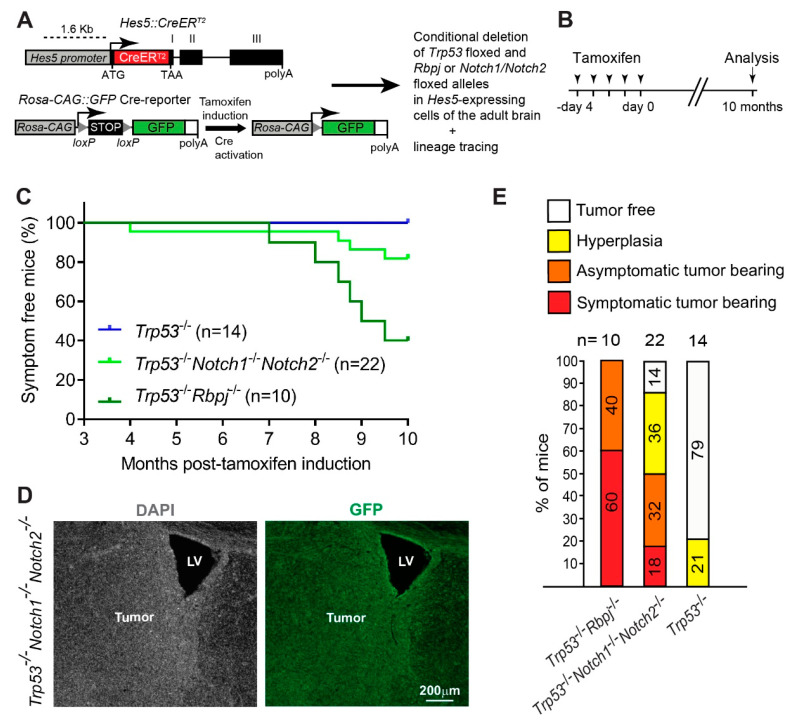
Combined *Notch1/Notch2* and *Trp53* deletion in Hes5^+^ cells leads to formation of forebrain tumors in adult mice. (**A**,**B**) Conditional deletion of *Notch1*, *Notch2* and *Trp53* or *Rbpj* and *Trp53* in brain cells expressing the Notch target gene *Hes5*. Adult *Hes5::CreER^T2^* mice carrying floxed *Trp53* alleles and either floxed *Notch1* and *Notch2* alleles or floxed *Rbpj* alleles and a GFP Cre-reporter for lineage tracing were treated with tamoxifen (**A**) and were analyzed 10 months later (**B**). (**C**) Kaplan–Meier curves showing survival of *Hes5::CreER^T2^ Trp53^-/-^*, *Trp53^-/-^Notch1^-/-^Notch2^-/-^* and *Trp53^-/-^Rbpj^-/-^* mutant mice. (**D**) A *Trp53^-/-^Notch1^-/-^Notch2^-/-^* tumor in the forebrain. The GFP expression from the *Rosa-CAG::GFP* Cre-reporter indicates derivation from Hes5^+^ cells. LV, lateral ventricle. (**E**) Percentages of *Trp53^-/-^*, *Trp53^-/-^Notch1^-/-^Notch2^-/-^* and *Trp53^-/-^Rbpj^-/-^* mice that developed hyperplasia or tumors in the brain.

To address whether simultaneous inactivation of the Notch1 and Notch2 receptors cooperates with p53 inactivation to promote tumor growth in the adult brain, we combined gene-ablation and long-term genetic lineage tracing. We used the *Hes5::CreER^T2^* transgenic mouse line to delete floxed *Trp53* alleles, either alone or in combination with floxed *Rbpj* or *Notch1*/*Notch2* alleles, specifically in Notch-active Hes5^+^ cells of the adult brain (Figure 1A) [13,16,28]. We have previously used the *Hes5::CreER^T2^* line to efficiently delete conditional alleles [12,18]. A Cre-reporter allele (Rosa-CAG::GFP) was used to lineage trace the cells (Figure 1A) [37]. We induced Cre-activity by Tamoxifen (TAM) treatment of 2-months-old young adult mice (Figure 1B). We then followed the animals for 10 months after TAM administration and harvested their brains at 1 year of age (Figure 1B). None of the *Trp53*^-/-^ mutant mice (*n* = 14) showed overt symptoms of tumor formation (Figure 1C). In contrast, and in agreement with our previous published data [28], 60% of the *Trp53*^-/-^*Rbpj*^-/-^ animals (*n* = 10) became symptomatic starting 7 months after induction with TAM (Figure 1C). Moreover, also 18% of the *Trp53*^-/-^*Notch1*^-/-^*Notch2*^-/-^ mutants (*n* = 22) developed symptoms and succumbed before 10 months to supratentorial brain tumors (Figure 1C,D). Histological examination showed that 79% of the *Trp53*^-/-^ mutant brains were normal and 21% showed localized hyperproliferation/hyperplasia (Figure 1E). In contrast, none of the *Trp53*^-/-^*Rbpj*^-/-^ animals and only 14% of the *Trp53*^-/-^*Notch1*^-/-^*Notch2*^-/-^ mutants had brains that appeared normal (Figure 1E). All *Trp53*^-/-^*Rbpj*^-/-^ mutants had brain tumors, and *Trp53*^-/-^*Notch1*^-/-^*Notch2*^-/-^ mutants had either tumor (50%) or hyperplasia (36%) (Figure 1E).

**Figure 2:**
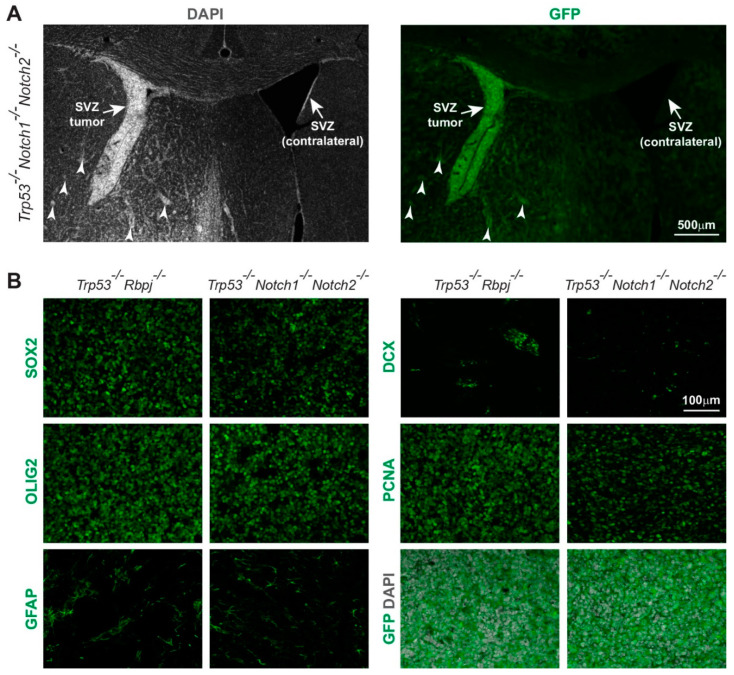
Immunohistochemical analysis of *Trp53^-/-^Notch1^-/-^Notch2^-/-^* and *Trp53^-/-^Rbpj^-/-^* tumors. (**A**) A *Trp53^-/-^Notch1^-/-^Notch2^-/-^* tumor in the SVZ. Streams of tumor cells invading the brain parenchyma are indicated by arrowheads. The GFP expression from the *Rosa-CAG::GFP* Cre-reporter indicates derivation from Hes5^+^ cells. (**B**) Expression of GFP, progenitor (SOX2) and glial markers (OLIG2, GFAP), as well as staining for mitotically active cells (PCNA) and immature neurons (DCX) in *Trp53^-/-^Notch1^-/-^Notch2^-/-^* and *Trp53^-/-^Rbpj^-/-^* tumors.

The expression of GFP by the tumor cells indicated derivation from Hes5^+^ cells (Figure 1D, Figure 2A,B and Figure S1A). *Trp53*^-/-^*Rbpj*^-/-^ and *Trp53*^-/-^*Notch1*^-/-^*Notch2*^-/-^ tumors showed similar expression of progenitor markers as well as markers of the oligodendrocyte (OLIG2), astrocyte (GFAP), and neuronal (DCX) lineages (Figure 2B and Figure S1B). Thus, loss of RBP-Jk and p53 or simultaneous loss of Notch1, Notch2 and p53 induced supratentorial brain tumors and hyperplasia formation with high penetrance.

### 3.2. Deletion of Notch1 and Trp53 in Hes5^+^ Cells Leads to the Formation of Forebrain Tumors in Adult Mice

**Figure 3:**
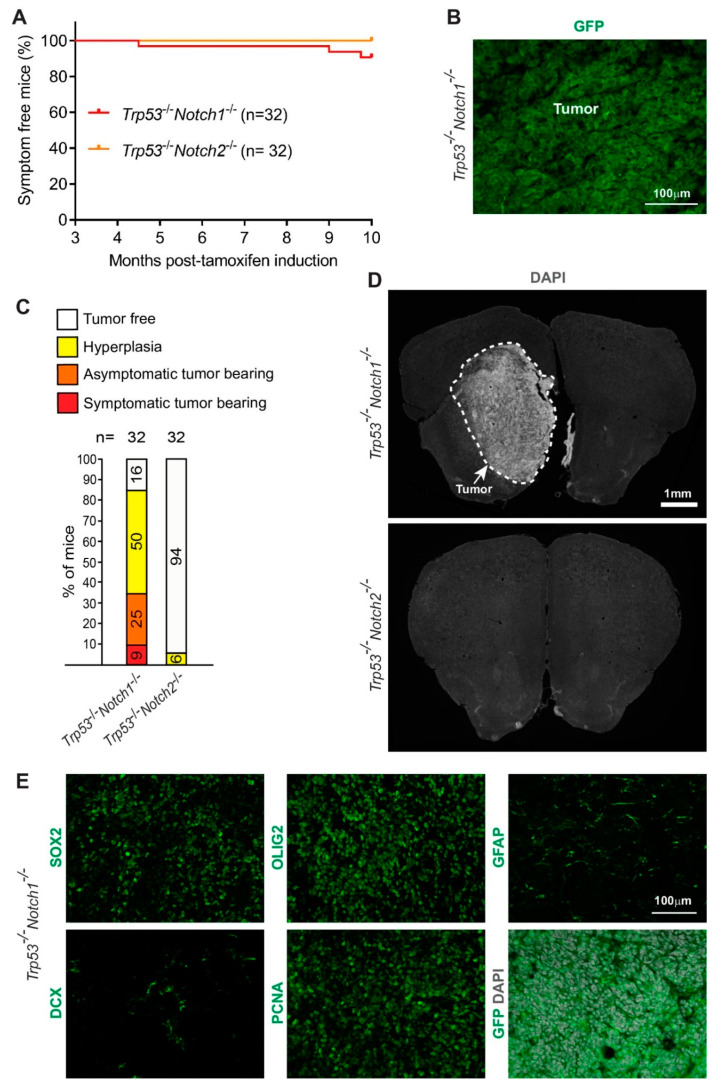
*Notch1* and *Trp53* deletion in Hes5^+^ cells leads to formation of forebrain tumors in adult mice. (**A**) Kaplan–Meier curves showing survival of *Hes5::CreER^T2^ Trp53^-/-^Notch1^-/-^* and *Trp53^-/-^Notch2^-/-^* mutant mice. (**B**) A *Trp53^-/-^Notch1^-/-^* tumor in the forebrain. The GFP expression from the *Rosa-CAG::GFP* Cre-reporter indicates derivation from Hes5^+^ cells. (**C**) Percentages of *Trp53^-/-^Notch1^-/-^* and *Trp53^-/-^Notch2^-/-^* mice that developed hyperplasia or tumors in the brain. (**D**) A *Trp53^-/-^Notch1^-/-^* tumor (dashed line) in the anterior forebrain. In contrast, the forebrain of *Trp53^-/-^Notch2^-/-^* mice appears grossly normal. (**E**) Expression of GFP, progenitor (SOX2) and glial markers (OLIG2, GFAP), as well as staining for mitotically active cells (PCNA) and immature neurons (DCX) in *Trp53^-/-^Notch1^-/-^* tumors.

The relative contribution of Notch1 and Notch2 receptors to the tumor-suppressive activity of Notch signaling in this context is unknown. We addressed whether loss of p53 in combination with loss of either Notch1 or Notch2 is tumorigenic in the adult brain. We induced Cre-activity by TAM treatment of floxed *Trp53/Notch1* and *Trp53/Notch2* adult mice carrying the *Hes5::CreER^T2^* transgene and followed the animals for 10 months (Figure 3). Overall, 9% of the *Trp53*^-/-^*Notch1*^-/-^ mutants (*n* = 32) showed symptoms of tumor formation during the chase period (Figure 3A). However, none of the *Trp53*^-/-^*Notch2*^-/-^ mice (*n* = 32) developed overt symptoms (Figure 3A). We examined the brains of *Trp53*^-/-^*Notch1*^-/-^ and *Trp53*^-/-^*Notch2*^-/-^ mice by histology. Overall, 34% of the *Trp53*^-/-^*Notch1*^-/-^ mutants had tumors, 50% had hyperplasia, and only 16% appeared normal (Figure 3B–D). In contrast, only 6% of the *Trp53*^-/-^*Notch2*^-/-^ mutant brains showed localized hyperproliferation/hyperplasia, and the remaining brains (94%) appeared normal (Figure 3C,D). Similar to the *Trp53*^-/-^*Notch1*^-/-^*Notch2*^-/-^ tumors (Figure 2B), *Trp53*^-/-^*Notch1*^-/-^ tumors expressed high levels of progenitor and oligodendrocyte lineage markers (SOX2, OLIG2), lower levels of astrocytic (GFAP) and neuronal (DCX) markers, and were highly proliferative (Figure 3E, Table 1). We concluded that *Notch1* and *Trp53* deletion in Hes5^+^ cells is sufficient to induce tumor or hyperplasia formation in the forebrains of adult mice.

### 3.3. Hyperplasia and Tumors Are Preferentially Located in Ventral Forebrain Regions and OBs

**Figure 4:**
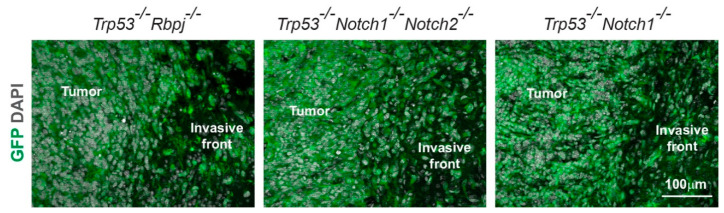
Pictures of the invasive front of *Trp53^-/-^Rbpj^-/-^*, *Trp53^-/-^Notch1^-/-^Notch2^-/-^* and *Trp53^-/-^Notch1^-/-^* tumors. The GFP expression from the *Rosa-CAG::GFP* Cre-reporter indicates derivation from Hes5^+^ cells.

**Figure 5:**
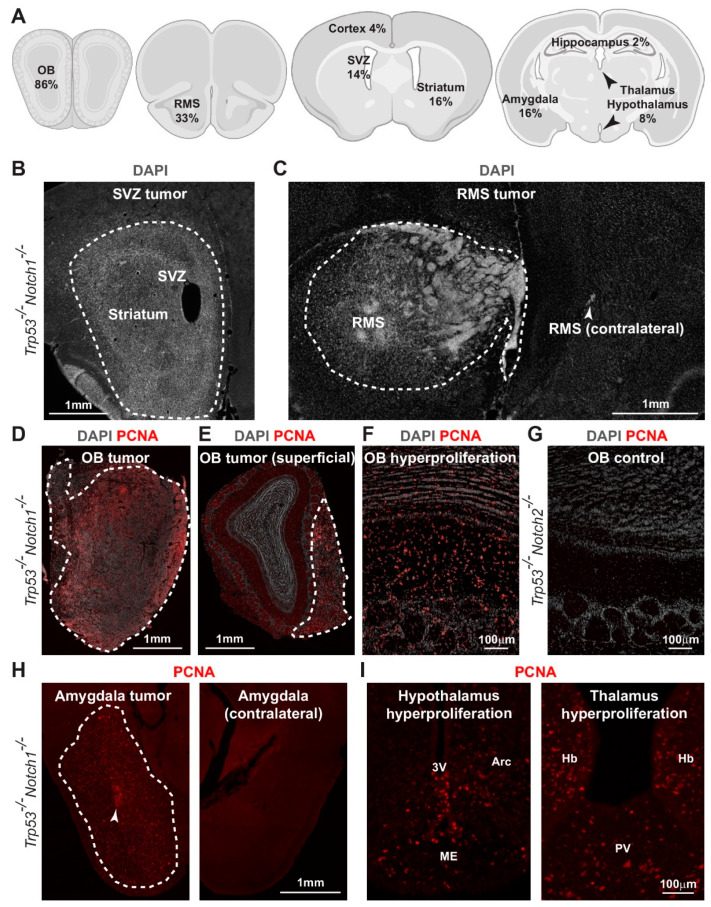
Hyperplasia and tumors are preferentially located in ventral forebrain regions and olfactory bulbs. (**A**) Representative schemes of sections of the mouse brain at different anteroposterior levels summarizing the distribution of hyperplasia and tumors. Percentages of mice with hyperplasia or tumors in a given location is indicated (see also Supplementary Table S1). (**B**) A *Trp53^-/-^Notch1^-/-^* tumor (dashed line) in the SVZ and adjacent striatum. (**C**) A *Trp53^-/-^Notch1^-/-^* tumor (dashed line) in the RMS and adjacent brain parenchyma. The contralateral RMS is indicated by the arrowhead. (**D**) A *Trp53^-/-^Notch1^-/-^* tumor (dashed line) that entirely occupies the OB. (**E**) A *Trp53^-/-^Notch1^-/-^* tumor (dashed line) located on the surface of the OB. (**F**) A diffuse, proliferating (PCNA) *Trp53^-/-^Notch1^-/-^* hyperplasia that invades all OB layers. (**G**) No signs of hyperplasia in the OB of a *Trp53^-/-^Notch2^-/-^* mouse. (**H**) A *Trp53^-/-^Notch1^-/-^* tumor (dashed line) in the amygdala region. Note the denser core of the tumor located in the lateral nucleus of the amygdala (arrowhead). The contralateral, tumor-free amygdala is also shown. (**I**) PCNA^+^ hyperplasia in the hypothalamus and thalamus of a *Trp53^-/-^Notch1^-/-^* mouse. 3V, 3rd ventricle; Arc, arcuate nucleus; ME, median eminence; Hb, habenular nucleus; PV, periventricular thalamic nucleus.

Examination of the tumor border showed moderate invasion of *Trp53*^-/-^*Rbpj*^-/-^*, Trp53*^-/-^*Notch1*^-/-^*Notch2*^-/-^, and *Trp53*^-/-^*Notch1*^-/-^ tumor cells into the surrounding brain parenchyma (Figure 4). All tumors that we identified had a supratentorial location, in agreement with our previous results from *Trp53*^-/-^*Rbpj*^-/-^ mutants [28]. However, the large size of *Trp53*^-/-^*Rbpj*^-/-^ tumors often precluded a more precise characterization of their location. We took advantage of the relatively smaller size of *Trp53*^-/-^*Notch1*^-/-^*Notch2*^-/-^, *Trp53*^-/-^*Notch1*^-/-^, and *Trp53*^-/-^*Notch2*^-/-^ tumors and hyperplasias compared to *Trp53*^-/-^*Rbpj*^-/-^ tumors to better define the prevalent sites of tumor formation within the forebrain after genetic inhibition of Notch and p53 (Figure 5, Table S1). We scored the number and location of tumors and hyperplasias in *Trp53*^-/-^*Notch1*^-/-^*Notch2*^-/-^, *Trp53*^-/-^*Notch1*^-/-^, *Trp53*^-/-^*Notch2*^-/-^, and *Trp53*^-/-^ mutants. Tumors and hyperplasias were predominantly located in ventral and anterior forebrain regions, including the OBs, the rostral migratory stream (RMS) and surrounding areas, and the subventricular zone (SVZ) and adjacent striatum (Figure 5A–G, Table S1). The OB was the brain region where malignant cells were most frequently identified (Figure 5A, Table S1). OB tumors and hyperplasias in *Trp53*^-/-^*Notch1*^-/-^*Notch2*^-/-^ and *Trp53*^-/-^*Notch1*^-/-^ animals were either large and invaded all OB layers (Figure 5D,F) or were superficial and preferentially occupied the glomerular and nerve layers (Figure 5E). The OBs of most *Trp53*^-/-^*Notch2*^-/-^ animals did not show signs of hyperproliferation and looked normal (Figure 5G). However, the rare, small hyperplasias that could be detected in *Trp53*^-/-^*Notch2*^-/-^ and *Trp53*^-/-^ mutants were also preferentially located in the OBs (Table S1). In contrast to the anterior and ventral forebrain, tumor formation very rarely affected dorsal forebrain regions such as the cerebral cortex and the hippocampus (Figure 5A, Table S1). Large tumors and hyperplasias were also relatively common in the amygdala region (Figure 5H, Table S1). In addition, hyperproliferation was observed in the hypothalamus and thalamus of a proportion of the mice (Figure 5I, Table S1). In some animals, multiple tumors and hyperplasias co-occurred at distant locations in the brain, for example OB and amygdala, OB and hypothalamus/thalamus, and OB and SVZ (Table S1).

## 4. Discussion

Our gene-ablation and long-term genetic lineage tracing experiments in mice indicate that Notch/RBP-Jk signaling, and particularly the Notch1 receptor, can inhibit brain tumor initiation and growth in the adult forebrain. *Trp53* deletion alone in cells expressing the Notch target gene *Hes5* predisposes to hyperplasia formation with very low penetrance. We have previously shown that deletion of *Rbpj*, *Notch1*, or *Notch2* in the presence of an intact *Trp53* gene alters NSC proliferation and differentiation, but does not induce formation of brain tumors [12,28]. In contrast, simultaneous deletion of *Trp53* and *Rbpj*, *Notch1/Notch2*, or *Notch1* induces tumor or hyperplasia formation in over 80% of the mice. Thus, Notch signaling cooperates with p53 to inhibit tumor formation in the adult murine brain. These data confirm and extend our previous findings from PDGF-driven gliomas and indicate that Notch signaling can behave as a tumor suppressor in the brain even in the absence of PDGF overexpression [28]. Although *Trp53*^-/-^*Rbpj*^-/-^ tumors are clearly separable from PDGF^+^*Trp53*^-/-^*Rbpj*^-/-^ tumors at the transcriptional level [28], it is conceivable that tumor cells in both models acquire oligodendrocyte progenitor cell (OPC)-like features, a cellular state that is associated with a proneural glioma transcriptional subtype and a tumor suppressor role of Notch signaling [20]. This scenario is in agreement with a previous study showing that the transcriptional profiles of murine glioma models driven by different oncogenic alterations can convergence to a proneural signature [38].

RBP-Jk can act as a transcriptional repressor and loss of the *RBPJ* gene promotes the growth of human breast cancer cells by de-repressing target gene promoters in a Notch-independent manner [39,40]. However, our data showing that not only *Trp53*^-/-^*Rbpj*^-/-^ mutants, but also *Trp53*^-/-^*Notch1*^-/-^*Notch2*^-/-^ and *Trp53*^-/-^*Notch1*^-/-^ mutants develop tumors suggest that the tumor suppressor function of RBP-Jk in the brain is linked to the regulation of Notch signaling. This hypothesis is supported by an in vivo CRISPR screen that identified *Notch1* among potential tumor suppressors in glioma [41], and is further in line with the occurrence of *NOTCH1*, *NOTCH2*, and *RBPJ* inactivating mutations and/or reduced Notch signaling activity in human glioma subtypes [21,23,26,27,28,29,30,31].

We found that *Trp53*^-/-^*Notch2*^-/-^ mutants rarely develop hyperplasia, in contrast to *Trp53*^-/-^*Notch1*^-/-^ and *Trp53*^-/-^*Rbpj*^-/-^ mutants. We cannot exclude that differences in recombination efficiency of *Notch1*, *Notch2*, and *Rbpj* conditional alleles contribute to the different penetrance of the phenotypes. However, we have recently shown that glioma cells lacking *Notch1* are more aggressive than glioma cells lacking *Notch2*, despite efficient deletion of both *Notch1* and *Notch2* floxed alleles [20]. Thus, our current finding confirms our observation that Notch1 is a stronger tumor suppressor than Notch2 in a PDGF-driven mouse model of glioma [20] and implies that Notch1 could partially compensate for Notch2 loss. Although *Notch2* deletion does not accelerate hyperplasia formation in *Trp53*^-/-^ mutants, it significantly accelerates the growth of PDGF^+^*Trp53*^-/-^ gliomas [20], suggesting that a strong mitogenic stimulus, provided, for instance, by PDGF, is required to release the tumorigenic effects of Notch2 inhibition. Our data do not exclude the residual expression of *Hes/Hey* gene family members or other Notch downstream targets, particularly in cells deleted for one Notch receptor, as a result of compensatory mechanisms or direct induction of *Hes* genes expression by BMP signaling [42]. In agreement with this hypothesis, we have recently shown that *Notch1* or *Notch2* deletion in PDGF^+^*Trp53*^-/-^ gliomas leads to changes in the expression of only partially overlapping sets of genes [20]. We observed tumor or hyperplasia formation in roughly 85% of *Trp53*^-/-^*Notch1*^-/-^*Notch2*^-/-^ and *Trp53*^-/-^*Notch1*^-/-^ mutants, but complete penetrance in *Trp53*^-/-^*Rbpj*^-/-^ mutants. This suggests that other receptors beyond Notch1 and Notch2 might be involved. It would be interesting to determine whether Notch3, which represses the proliferation of adult NSCs [43,44], contributes to the tumor suppressor activity of RBP-Jk in cooperation with Notch1 and Notch2.

We found that a proportion of the tumors and hyperplasias were located in the proximity of neurogenic regions including the RMS and SVZ and adjacent striatum, implying that adult neural stem and progenitor cells are probable cells of origin of some of the tumors [45]. However, a significant proportion of the tumors and hyperplasias were confined to anterior and ventral brain regions such as the surface of the OB, the amygdala, the hypothalamus and the thalamus, raising the possibility that some of the tumors originate locally. Parenchymal OPCs express Notch receptors [46,47,48] and can be an important cell type of origin of tumors, notably in the anterior and ventral brain [49,50,51,52]. Further work is needed to explore whether the resilience of ventral OPCs to pathological stimuli can make them exceptionally vulnerable to malignant transformation [49,53]. It is also possible that the glycosylation status of the Notch receptors, which can affect ligand binding, as well as varying expression levels of the Notch ligands contribute to regional heterogeneity in tumorigenesis [54,55]. In the future, it would be interesting to determine whether the frequency of tumor formation correlates with domain specific expression of Jagged and Delta ligands in different brain areas.

We show that genetic inactivation of Notch signaling and p53 in a subpopulation of brain cells induces CNS tumors with long latency but high penetrance. Notch-regulated competitive interactions, triggered by dishomogeneus Notch inactivating mutations in adjacent cells, contribute to the initiation of epithelial cancers by favoring the slow and progressive expansion of mutant clones at the expense of wild-type cells [56]. Intriguingly, data suggest that transformed NSCs can outcompete neighboring NSCs by repressing their proliferation through Notch signaling [57]. Whether Notch inhibition in malignant NSCs exacerbates cell competition and fitness selection is unknown. Likewise, OPC populations compete for space in the developing brain [58], but whether a similar phenomenon occurs during glioma formation from OPCs remains unclear. We speculate that Notch-regulated cell competition mechanisms could be in place during tumor initiation and progression in the brain.

## 5. Conclusions

In conclusion, we show that *Notch1* deletion cooperates with *Trp53* deletion to promote tumor growth in the adult forebrain. Our data also suggest that tumors induced by Notch signaling inhibition have a multifocal origin. These findings shed light on the pleiotropic roles of Notch signaling in the development of primary brain malignancies.

## Figures and Tables

**Table 1:** PCNA^+^ cells (% of GFP^+^ cells) in hyperplasias and tumors. Data represent mean ± standard deviation.

	*Trp53* ^-/-^	*Trp53* ^-/-^ *Rbpj* ^-/-^	*Trp53* ^-/-^ *Notch1* ^-/-^ *Notch2* ^-/-^	*Trp53* ^-/-^ *Notch1* ^-/-^	*Trp53* ^-/-^ *Notch2* ^-/-^
**Hyperplasia**	49.5 ± 10.5 (*n* = 3)	n/a	73.6 ± 4.3 (*n* = 5)	63.7 ± 9.1 (*n* = 5)	55.0 ± 3.8 (*n* = 2)
**Tumor**	n/a	89.6 ± 0.6 (*n* = 4)	85.4 ± 4.9 (*n* = 4)	76.7 ± 5.5 (*n* = 5)	n/a

## Data Availability

All data generated or analyzed during this study are included in this article and its Supplementary Material files. Further enquiries can be directed to the corresponding author.

## Supplementary Materials

The following supporting information can be downloaded at: https://www.mdpi.com/article/10.3390/cancers14215409/s1, Table S1: Distribution of hyperplasia and tumors. Figure S1: Immunohistochemical analysis of *Trp53^-/-^Rbpj^-/-^* tumors and contralateral hemispheres.
