# Supplementary material for: Genetic Inactivation of Notch1 Synergizes with Loss of Trp53 to Induce Tumor Formation in the Adult Mouse Forebrain
Source: Cancers (Basel). 2022 Nov 2;14(21):5409. doi: 10.3390/cancers14215409 (PMC9655454; doi:10.3390/cancers14215409)
Supplement: Supplementary file 1 [file cancers-14-05409-s001.zip › Figure S1 and legend.pdf]

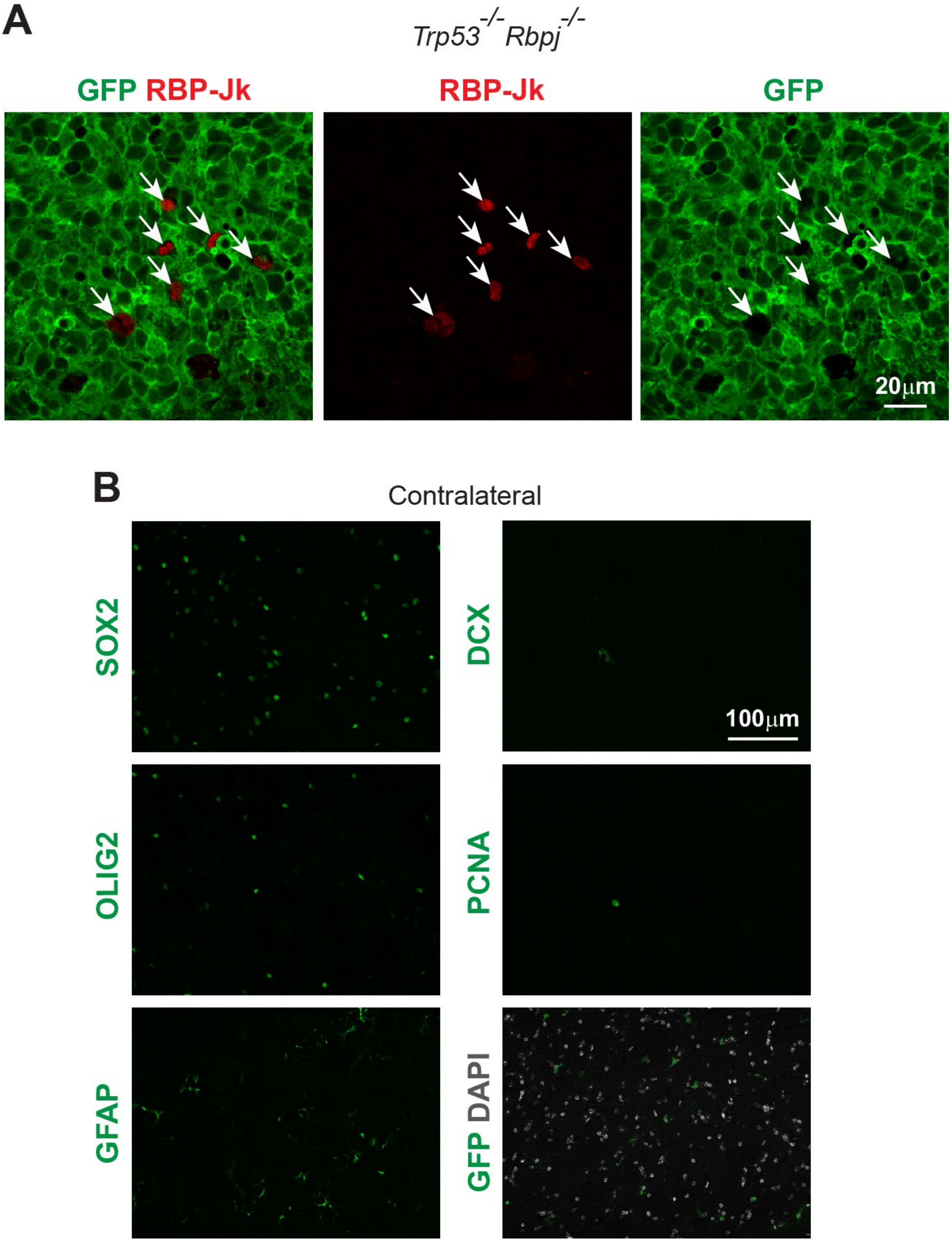

Figure S1. Immunohistochemical analysis of *Trp53*$^{-/-}$*Rbpj*$^{-/-}$ tumors and contralateral hemispheres. (**A**) Expression of RBP-Jk in a *Trp53*$^{-/-}$*Rbpj*$^{-/-}$ tumor. Note that RBP-Jk^{+} cells are GFP^{-} (arrows), and GFP^{+} tumor cells do not express RBP-Jk. (**B**) Expression of GFP, progenitor and glial markers (SOX2, OLIG2, GFAP), as well as staining for mitotically active cells (PCNA) and immature neurons (DCX) in the hemisphere contralateral to a *Trp53*$^{-/-}$*Rbpj*$^{-/-}$ tumor.
